# Supplementary material for: Comparative Study of CoFe2O4 Nanoparticles and CoFe2O4-Chitosan Composite for Congo Red and Methyl Orange Removal by Adsorption
Source: Nanomaterials (Basel). 2021 Mar 12;11(3):711. doi: 10.3390/nano11030711 (PMC8001270; doi:10.3390/nano11030711)
Supplement: Supplementary file 1 [file nanomaterials-11-00711-s001.pdf]

Supplementary Material

Comparative Study of $CoFe_2O_4$ Nanoparticles and $CoFe_2O_4$-Chitosan Composite for Congo Red and Methyl Orange Removal by Adsorption

Claudia Maria Simonescu[1,*], Alina Tătăruş[1,2], Daniela Cristina Culiţă[3,*], Nicolae Stănică[3], Ioana Alexandra Ionescu[2], Bogdan Butoi[4], Ana-Maria Banici[4]

[1]Politehnica University of Bucharest, Faculty of Applied Chemistry and Materials Science, Department of Analytical Chemistry and Environmental Engineering, Polizu Street, No.1-7, District 1, 011061, Bucharest; claudiamaria_simonescu@yahoo.com (C.M.S.); alina.tatarus@yahoo.com (A.T.);
[2]National Research and Development Institute for Industrial Ecology, INCD ECOIND Bucuresti, 71-73 Drumul Podul Dambovitei Str., 060652, Bucharest, Romania; alina.tatarus@yahoo.com (A.T.); ioana.ionescu@incdecoind (I.A.I.);
[3]Ilie Murgulescu Institute of Physical Chemistry, 202 Splaiul Independentei, 060021, Bucharest, Romania; danaculita@yahoo.co.uk (D.C.C.); nstanica@icf.ro (N.S.);
[4] National Institute for Laser, Plasma and Radiation Physics, Măgurele 077125, Romania; bogdan.butoi@inflpr.ro (B.B.); niculescu.anam@gmail.com (A.M.B);

*Correspondence: claudiamaria_simonescu@yahoo.com (C.M.S.); Tel.: (+40753071418); danaculita@yahoo.co.uk (D.C.C.); Tel: (+40765309363)

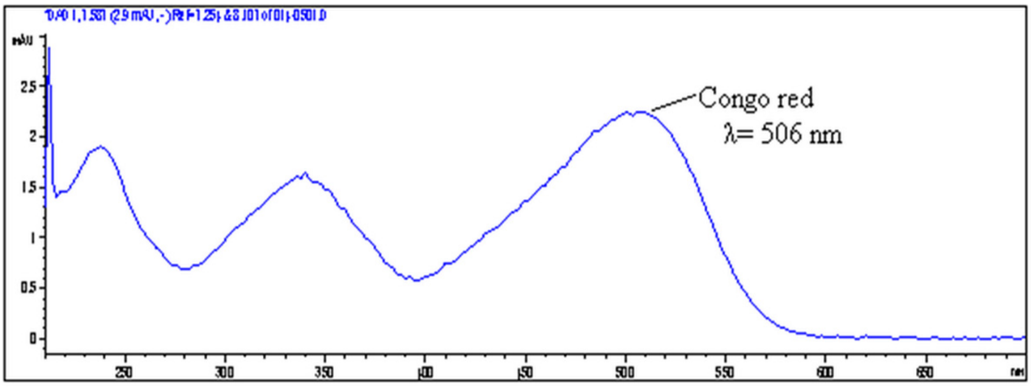

Figure S1. UV-VIS absorption spectra of CR obtained by HPLC-DAD

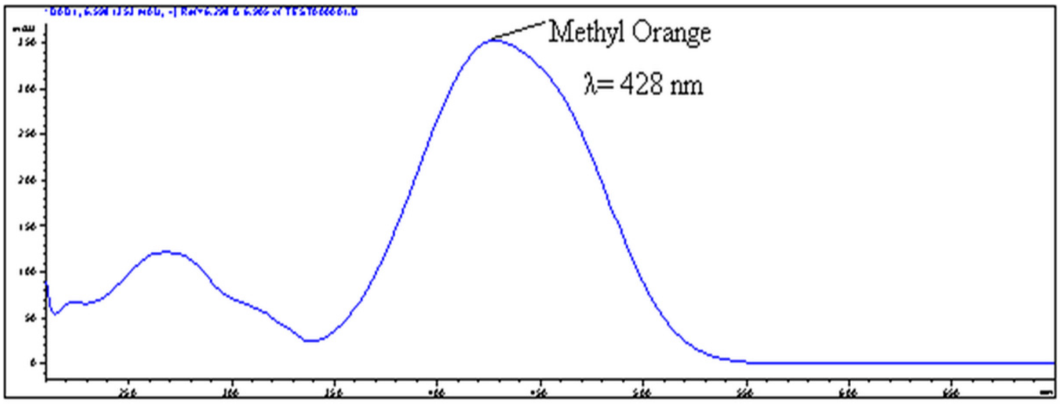

Figure S2. UV-VIS absorption spectra of Methyl Orange obtained by HPLC-DAD

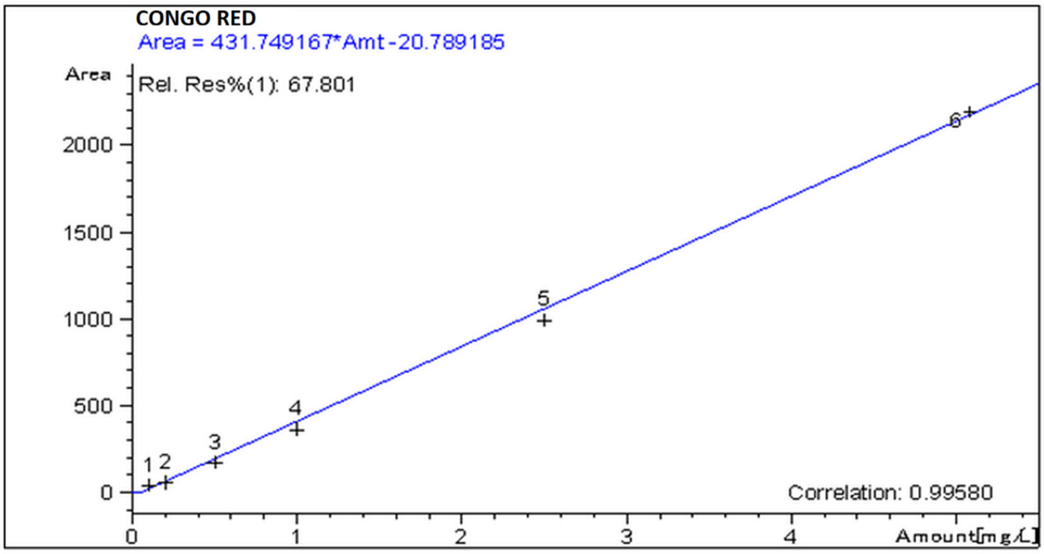

Figure S3. HPLC-DAD calibration curve of Congo Red

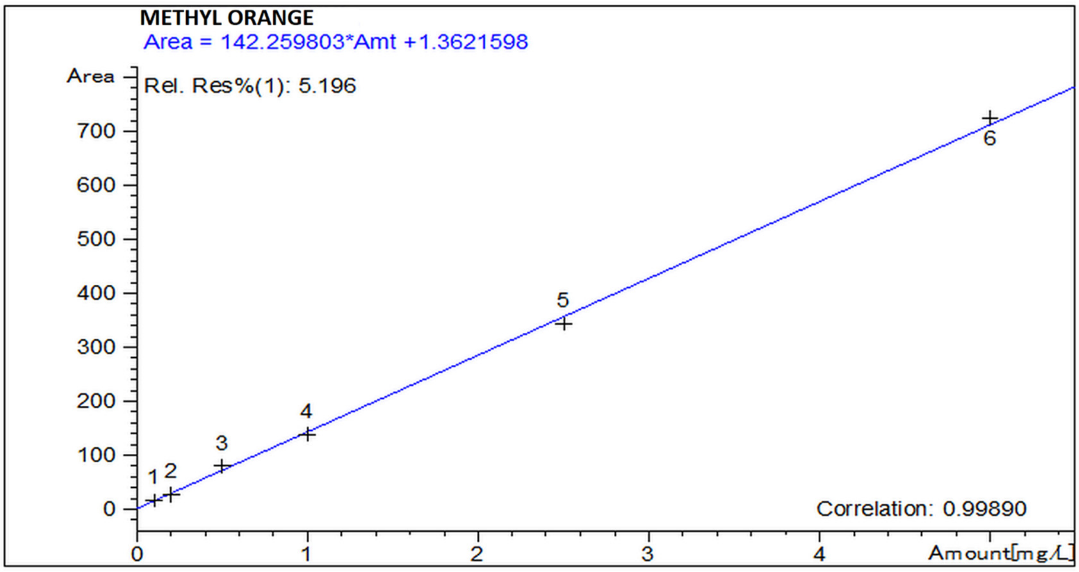

Figure S4. HPLC-DAD calibration curve of Methyl Orange

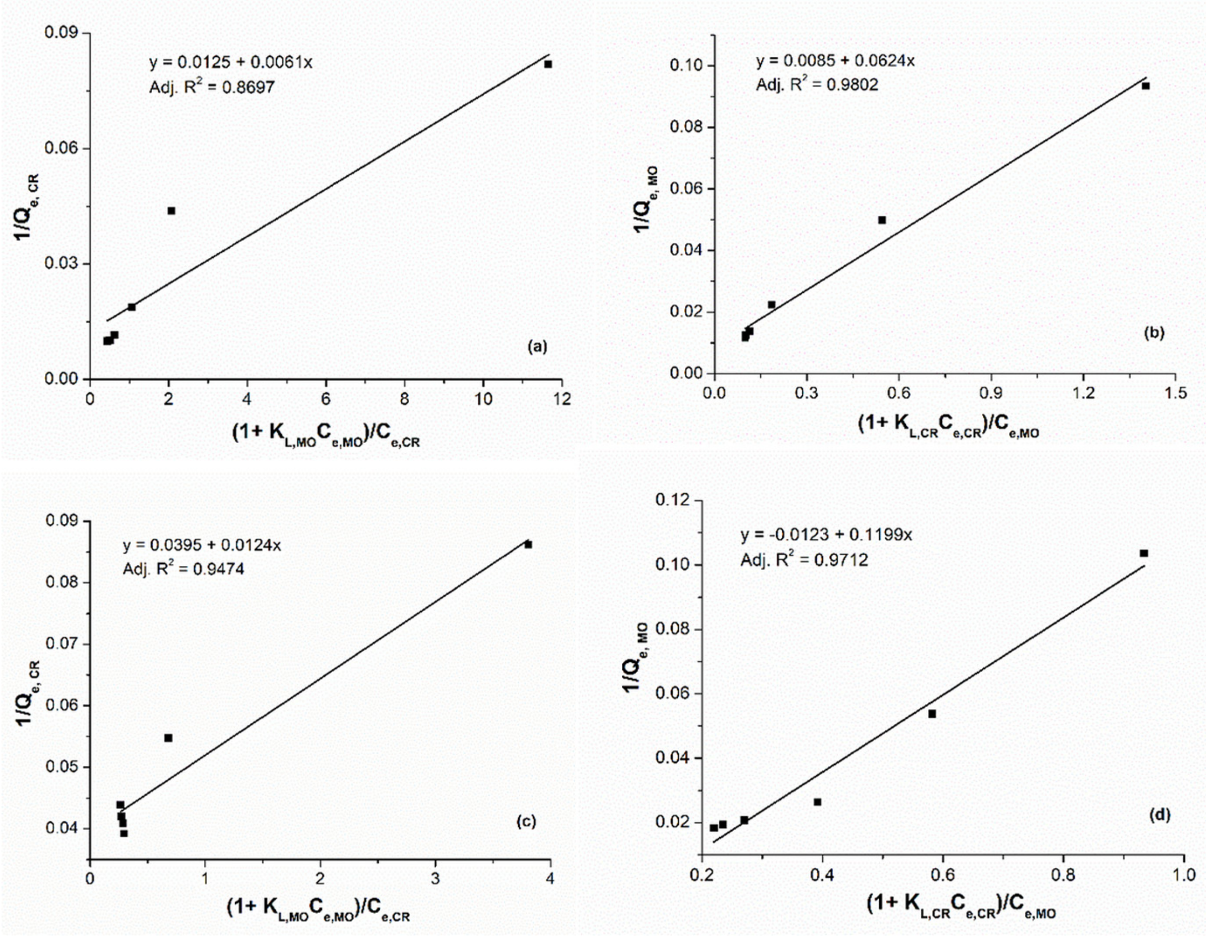

Figure S5. The modified Langmuir isotherm model for (a) CR in binary solution (CR + MO) and (b) MO in binary solution (CR + MO)

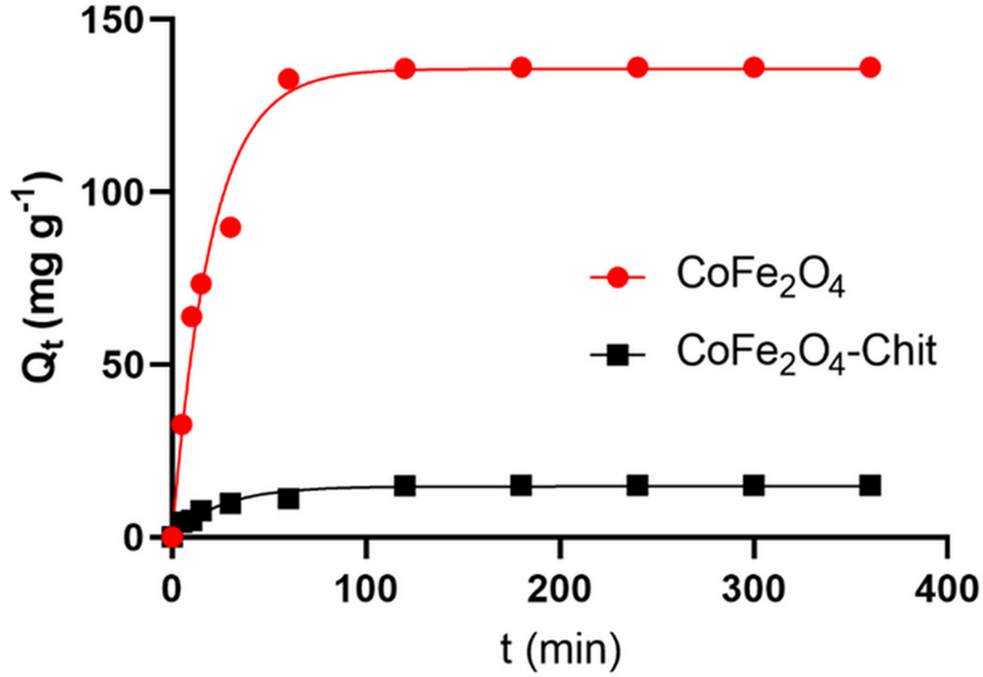

Figure S6. Graphical representation of the pseudo-first order kinetic model for removal of CR by adsorption on $CoFe_2O_4$ and $CoFe_2O_4$-Chit (nonlinear regression)

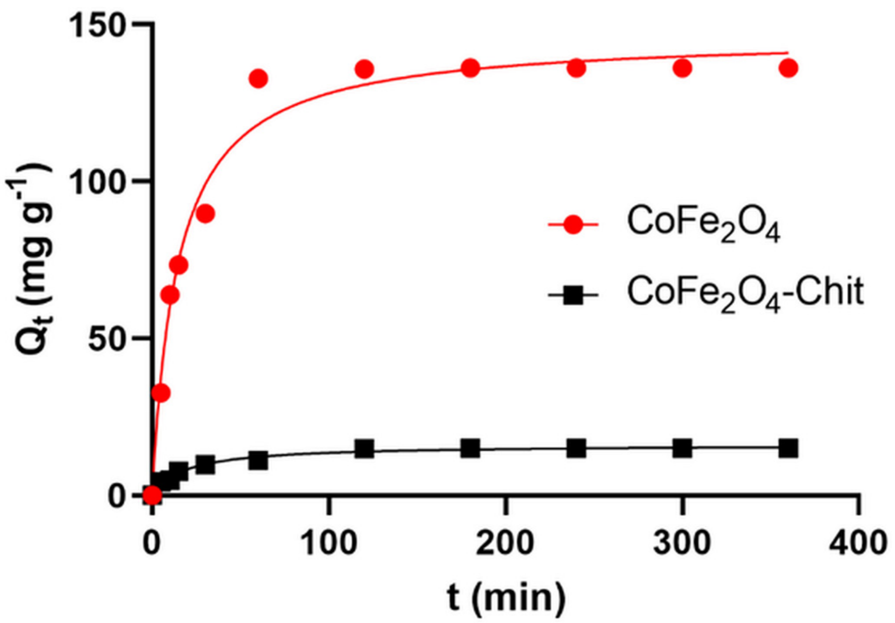

Figure S7. Graphical representation of the pseudo-second order kinetic model for removal of CR by adsorption on $CoFe_2O_4$ and CoFe2O4-Chit (nonlinear regression)

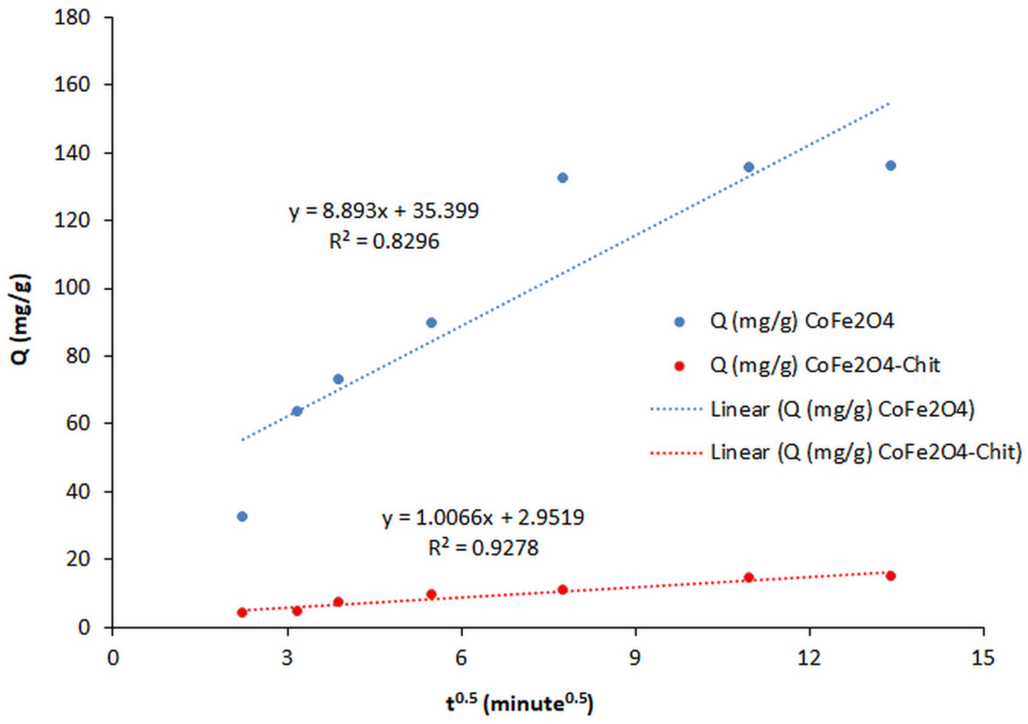

Figure S8. Graphical representation of the intraparticle diffusion model for the process of removal of CR by adsorption on $CoFe_2O_4$ and $CoFe_2O_4$-Chit

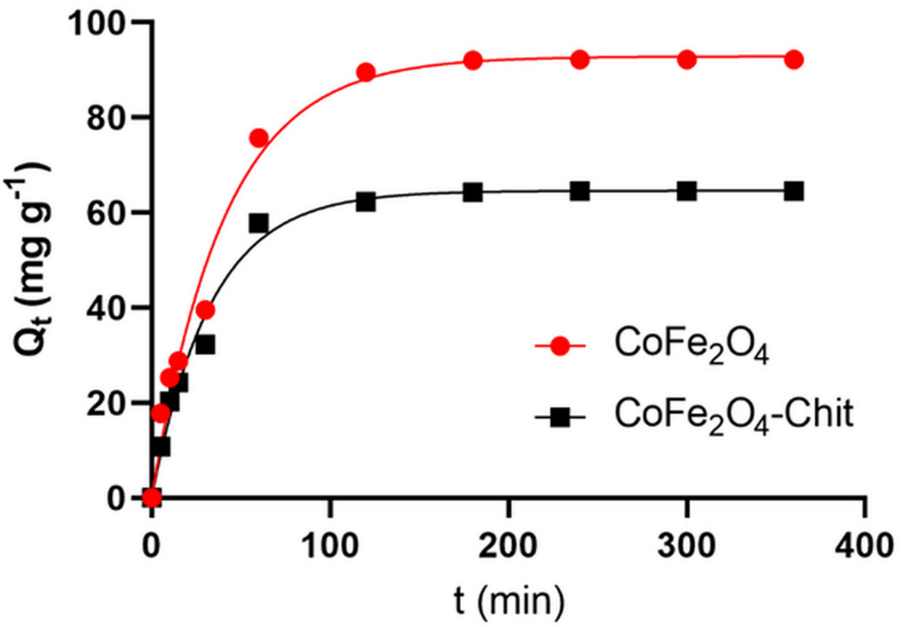

Figure S9. Graphical representation of the pseudo-first order kinetic model for removal of MO by adsorption on $CoFe_2O_4$ and $CoFe_2O_4$-Chit (nonlinear regression)

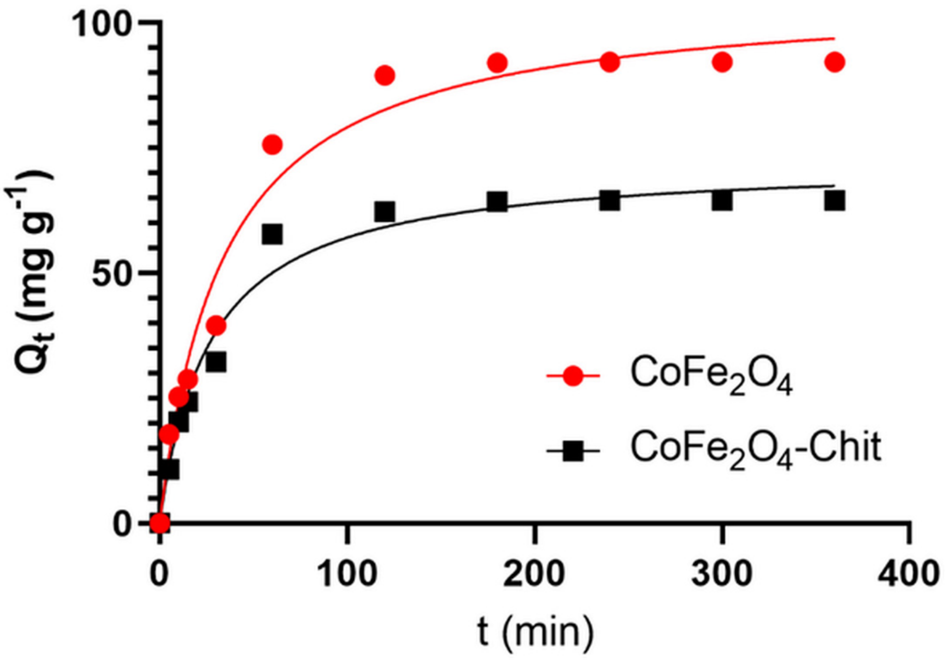

Figure S10. Graphical representation of the pseudo-second order kinetic model for removal of MO by adsorption on $CoFe_2O_4$ and CoFe2O4-Chit (nonlinear regression)

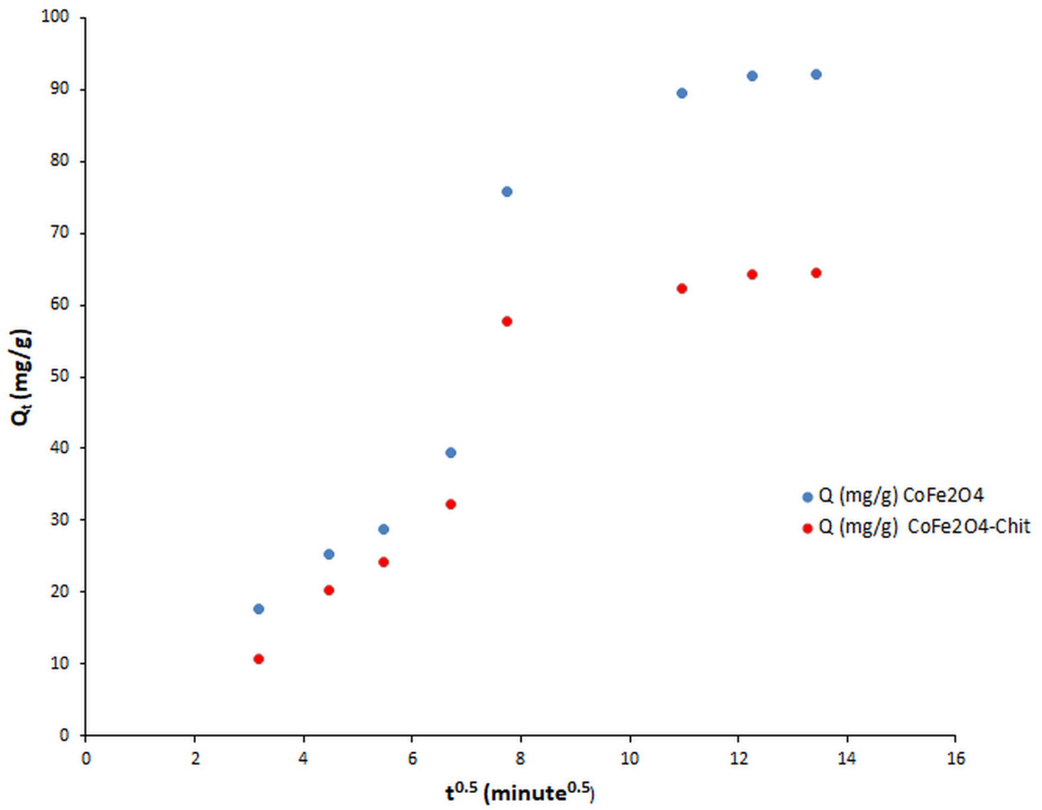

Figure S11. Graphical representation of the intraparticle diffusion model for the process of removal of MO by adsorption on $CoFe_2O_4$ and $CoFe_2O_4$-Chit

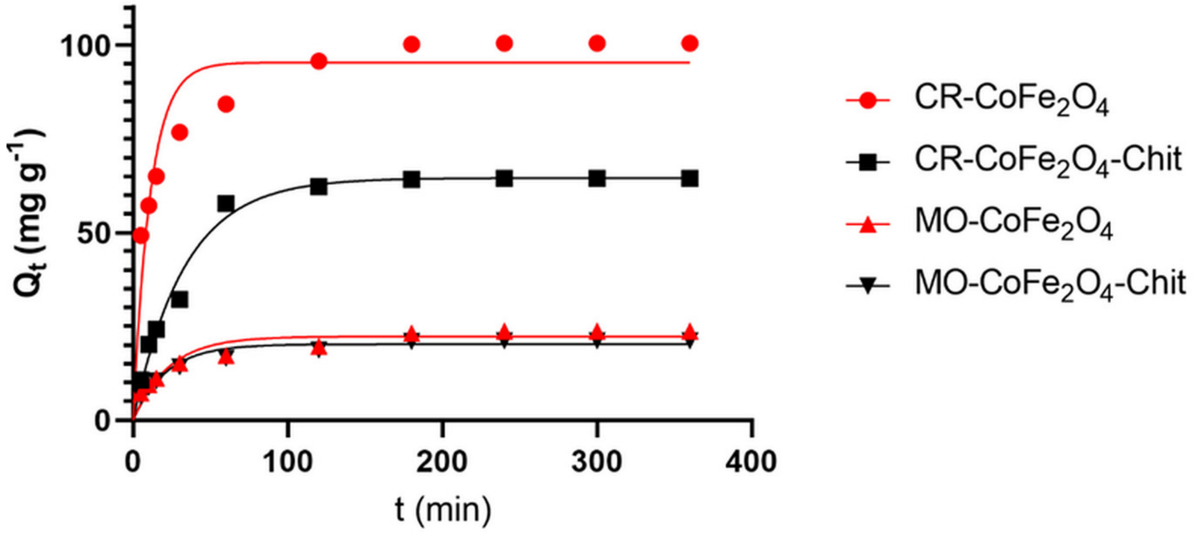

Figure S12. Graphical representation of the pseudo-first order kinetic model for removal of CR and MO by adsorption on $CoFe_2O_4$ and $CoFe_2O_4$-Chit from binary dye solutions (nonlinear regression)

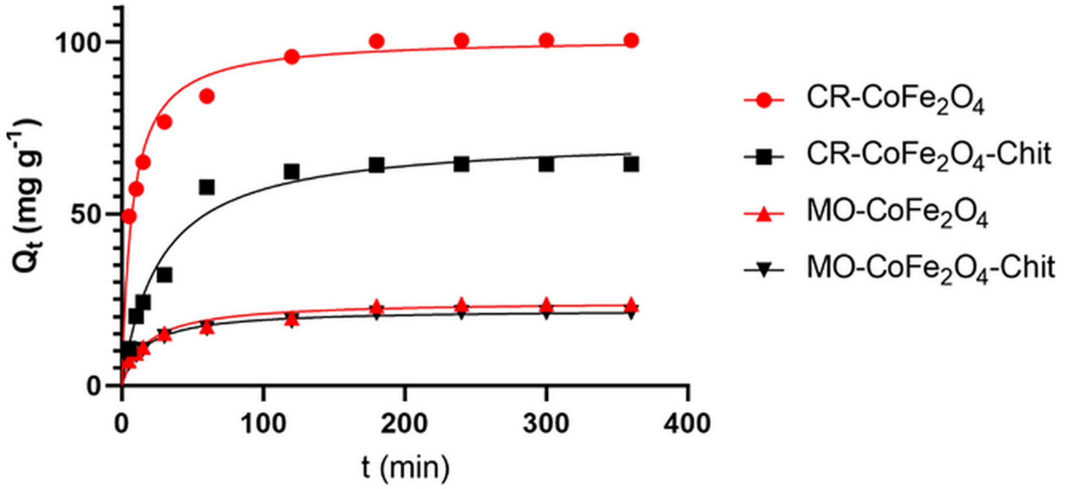

Figure S13. Graphical representation of the pseudo-second order kinetic model for removal of CR and MO by adsorption on $CoFe_2O_4$ and $CoFe_2O_4$-Chit from binary dye solutions (nonlinear regression)

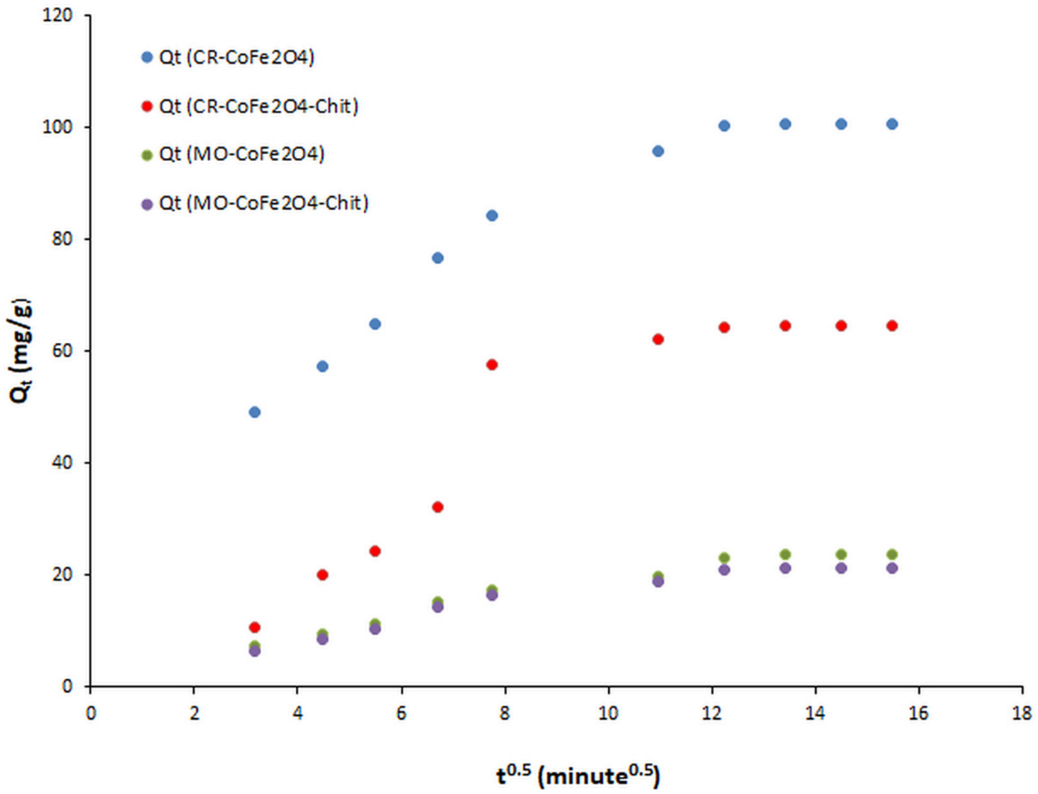

Figure S14. Graphical representation of the intraparticle diffusion kinetic model for removal of CR and MO by adsorption on $CoFe_2O_4$ and $CoFe_2O_4$-Chit from binary dye solutions
